# Metabolism and growth adaptation to environmental conditions in *Drosophila*

**DOI:** 10.1007/s00018-020-03547-2

**Published:** 2020-05-24

**Authors:** Takashi Koyama, Michael J. Texada, Kenneth A. Halberg, Kim Rewitz

**Affiliations:** Department of Biology, University of Copenhagen, Copenhagen, Denmark

**Keywords:** *Drosophila*, Insulin, Adipokinetic hormone, Metabolism, PTTH, Ecdysone

## Abstract

Organisms adapt to changing environments by adjusting their development, metabolism, and behavior to improve their chances of survival and reproduction. To achieve such flexibility, organisms must be able to sense and respond to changes in external environmental conditions and their internal state. Metabolic adaptation in response to altered nutrient availability is key to maintaining energy homeostasis and sustaining developmental growth. Furthermore, environmental variables exert major influences on growth and final adult body size in animals. This developmental plasticity depends on adaptive responses to internal state and external cues that are essential for developmental processes. Genetic studies have shown that the fruit fly *Drosophila*, similarly to mammals, regulates its metabolism, growth, and behavior in response to the environment through several key hormones including insulin, peptides with glucagon-like function, and steroid hormones. Here we review emerging evidence showing that various environmental cues and internal conditions are sensed in different organs that, via inter-organ communication, relay information to neuroendocrine centers that control insulin and steroid signaling. This review focuses on endocrine regulation of development, metabolism, and behavior in *Drosophila*, highlighting recent advances in the role of the neuroendocrine system as a signaling hub that integrates environmental inputs and drives adaptive responses.

## Introduction

Organisms must adapt to changing environments by adjusting their developmental growth, metabolism, and behavior to promote survival and reproduction. This adaptation relies on the ability to sense and respond to changes in internal and external environmental conditions. This involves complex sensing of nutritional conditions, temperature, oxygen, and light. Animals at all developmental stages integrate this information and adjust their metabolism and behavior to take advantage of available resources and to maintain homeostasis. Furthermore, juvenile animals—those that are still in the non-reproductive growth phase of their development—adjust their growth and development to meet resource availability in such a way that the final adult animal is most likely to be reproductively successful. The mechanisms that govern developmental, metabolic, and behavioral adaptations frequently make use of systemic endocrine signals to adjust the parameters of underlying genetic programs that control growth, developmental transitions, and physiology. This review explores endocrine mechanisms of environmental adaptation in the fruit fly *Drosophila melanogaster*, first investigating the modulation of growth and maturation during juvenile larval life and then investigating adult behavioral and metabolic adaptation. Environmental and internal inputs reflecting temperature, light, nutritional stores, food qualities (composition, odor, taste), and oxygen are covered, although others exist beyond the scope of this review such as humidity, CO_2_, and gut microbiota.

*Drosophila* has become an attractive model for understanding the endocrine regulation of growth and metabolic adaptation. Nutrients are digested and absorbed through the intestine, which is also a key endocrine organ that plays a central role in sensing nutritional information and relaying it to other tissues to maintain systemic metabolic homeostasis [1]. The *Drosophila* fat body and peripheral oenocytes serve the functions of the mammalian hepatic and adipose tissues [2, 3], both of which store energy (as glycogen and lipid, respectively) but also have endocrine function. In *Drosophila*, growth is restricted to larval stages called instars, and maturation is induced by reaching a critical size that triggers the onset of metamorphosis, which transforms the juvenile growing larva into the reproductive adult and largely limits any further growth [4, 5]. The larva can alter its growth rate and the duration of its growth period (determined by the timing of metamorphosis) to reach a final adult size that maximizes fitness and survival in variable environments. In nutrient-rich conditions, animals grow quickly and soon develop into adults. On the other hand, when nutrients are limited, the larval growth period is extended to allow additional growth and to ensure an appropriate final adult size under unfavorable growth conditions.

The main factors regulating growth and development according to the environment in animals are the conserved insulin and insulin-like growth factors (IGFs) and steroid hormones [6–8]. Work has shown that the *Drosophila* insulin-like peptides (DILPs) are the main regulators of tissue growth, whereas the steroid hormone ecdysone is the main factor that controls the duration of the growth period, although it also affects growth rate [9, 10]. The primary source of systemically acting growth-regulating DILPs is the population of so-called insulin-producing cells (IPCs) in the brain [11], thought to be equivalent to the mammalian pancreatic β cells [12]. The DILPs act in peripheral target tissues through a single insulin receptor (InR). Ecdysone is produced and released from the prothoracic gland (PG), a major endocrine organ, in response to DILPs and prothoracicotropic hormone (PTTH), another brain-derived neuropeptide [5, 13]. Developmental and environmental cues are integrated in the IPCs and PTTH-producing neurons (PTTHn) as well as by the PG itself to adjust insulin and ecdysone signaling according to intrinsic and extrinsic conditions, in order to adapt growth and development. These systems are all discussed in detail below.

Insulin/IGF signaling has two important roles: to regulate overall growth during development and to control metabolic homeostasis [14, 15]. As in mammals, circulating sugar levels and energy storage versus mobilization are regulated by the opposing effects of two hormones in *Drosophila*, insulin and Adipokinetic hormone (Akh, in some ways functionally analogous to mammalian glucagon). Following the intake of dietary sugar, insulin secretion promotes its tissue uptake from the hemolymph (insect circulatory fluid), whereas Akh induces mobilization of lipids and breakdown of glycogen to maintain hemolymph levels of lipids and sugars in response to starvation or exertion. In addition to these metabolic homeostatic circuits, regulation of food intake by modulation of appetite, odor and taste sensation, foraging, and food palatability is a major factor required for adaptation to nutritional conditions. Following prolonged deprivation of protein in their diet, flies preferentially select amino acid-rich food, based on certain taste neurons whose activity is regulated by the internal nutritional state [16]. On the other hand, deprivation of dietary sugars specifically increases feeding on sugar-rich foods. Feeding decisions are controlled by neuromodulators such as neuropeptides and hormones that change the motivational state according to the nutritional demand of the animal. In flies, these include the neuropeptide Diuretic hormone 44 (Dh44), an orthologue of the mammalian corticotropin-releasing hormone (CRH), which is involved in detecting the nutritional value of consumed sugars [17] and amino acids [18].

The mammalian hormone leptin provides an example of the useful parallels between fly and mammalian developmental endocrinology. Leptin, released from adipose cells in response to their lipid content (a reflection of nutritional state), modulates appetite and metabolism by signaling to the brain [19]. It furthermore regulates the activity of the neuroendocrine/steroid system that controls the onset of sexual maturation [20], which may explain the link between childhood obesity and early puberty. Flies possess a structurally and functionally similar hormone named Unpaired-2 (Upd2). Like leptin, *Drosophila* Upd2 is a nutrient-dependent adipokine that relays nutritional information to the brain [21]. Upd2 stimulates insulin secretion, which promotes growth and maturation onset through its effect on the production of the steroid hormone ecdysone [9, 22]. Thus, during development in both insects and mammals, endocrine signals related to the amount of body fat provide nutrient-status information to the neuroendocrine signaling system that initiates maturation. Here we will review some of the recent advance to highlight how inter-organ signaling networks allow *Drosophila* to adjust larval growth and development to variable environments, and we also examine endocrine mechanisms underlying metabolic and behavioral adaptations.

## Adaptation of larval growth and development

Animals reared in environments differing in temperature, oxygen level, and the availability of food develop at different rates into adults of different sizes. In nutritionally poor or low-oxygen (hypoxic) environments, *Drosophila* larvae grow slowly and attain a smaller adult body size, whereas in nutrient- and oxygen-rich environments, larvae develop more quickly into larger adults [23–27]. In contrast, low temperature also slows the growth of larvae and prolongs their development but results in increased adult body size [28], suggesting that temperature affects developmental growth by different mechanisms than oxygen and nutrients. Furthermore, changes in these environmental conditions affect the proportions of different body parts relative to the whole body [26, 29]. This developmental flexibility involves adaptive responses within the boundaries of species-specific genetic developmental frameworks to produce adults of sizes and proportions that suit prevailing environmental conditions. This developmental plasticity is regulated by nutrition-dependent hormonal signaling pathways that control tissue growth and feed into the endocrine system that determines the timing of metamorphosis and thus the length of the growth period.

**Fig. 1:**
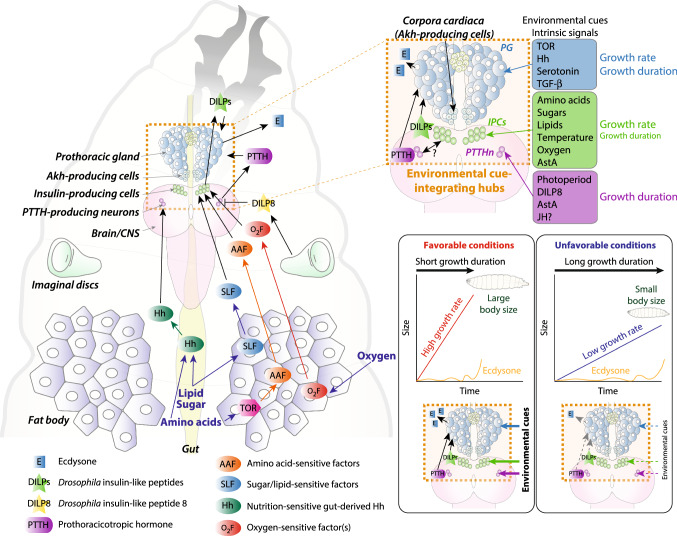
Growth-regulating environmental and internal cues are integrated through inter-organ communication in the *Drosophila* larva. In the main panel, larval organs communicate with one another via diffusible factors to govern growth and development. The upper right panel shows a magnified view of the larval central nervous system including the insulin-producing cells (IPCs) and PTTH-producing neurons (PTTHn) and the ring gland, which comprises the ecdysone-synthesizing prothoracic gland (PG), the Akh-producing cells (APCs) of the corpora cardiaca (CC), and the JH-producing corpora allata (between the lobes of the PG). Factors that act on growth and development via these various cells are indicated. The bottom-right schematic illustrates the relationships between size, growth rate, and growth duration

Steroid-hormone and insulin-like signaling pathways form the core axes of environmentally adaptive systemic regulation of growth and development in metazoans, and these pathways are thus evolutionarily ancient and have been conserved since before the divergence of flies and humans [6–8]. In *Drosophila*, DILPs (or insulin for simplicity), the steroid molting hormone 20-hydroxyecdysone (or "ecdysone" hereafter) and the sesquiterpenoid juvenile hormone (JH), as well as the intracellular nutrient-sensing Target of Rapamycin (TOR) pathway, are the principal regulators of growth rate and duration (Fig. 1). In this section, we review recent findings elucidating mechanisms by which larval signaling hubs integrate internal and external information and transduce it into growth-regulatory signals (insulin and ecdysone) that systemically control growth. In addition, we also discuss one of the most important environmentally sensitive checkpoints, named “critical weight,” which allows animals to adapt their growth period to different nutritional conditions, to reach an appropriate final body size. Finally, we propose a hypothesis that may explain how studying this checkpoint mechanism can potentially contribute to our understanding of human size regulation.

### Linking growth to nutrition, oxygen, and temperature through the DILP signaling pathway

Nutritional availability is a major environmental factor governing growth and development [30, 31]. Systemic DILP signaling and the cell-autonomous TOR pathway are the main mechanisms that regulate growth in response to nutrition. Because these pathways intersect with each other and share many downstream components, these pathways are often referred to jointly as insulin/TOR signaling. In *Drosophila*, high internal energy levels induces the activation of TOR in the fat body, which releases humoral factors to cause the IPCs to express and release various DILPs [11, 32]. Although the DILPs are differentially regulated by various stimuli, they act through the single InR. The primary systemically acting DILPs are DILP2, DILP3, and DILP5, expressed and released independently in response to nutritional conditions [11]. These DILPs are produced in bilateral clusters of neurosecretory cells—the IPCs—within the larval and adult medial protocerebrum [11], thought to be equivalent to the mammalian pancreatic β cells [12]. These cells send projections to neurohemal release sites near the esophageal foramen and, in the larva, to the PG, where they contribute to the regulation of ecdysone synthesis [9, 22]. Beyond the central IPCs, other tissues also produce DILPs for local or systemic signaling. For example, neuroblast growth within the nervous system is driven by local DILP production in glia, not from the IPCs [33, 34]. Furthermore, after the onset of metamorphosis, when larvae stop feeding, tissue growth is sustained through the secretion of DILP6 by the fat body [35, 36]. Thus, the pool of DILPs that mediate tissue growth is diverse in spatial and temporal expression.

Activation of InR by DILP binding results in a series of signaling events mediated by insulin receptor substrate (IRS; Chico in the fly) [37], phosphatidylinositide 3-kinase (PI3K or Dp110), and Akt (protein kinase B) [38]. One of the primary Akt targets is the transcription factor Forkhead Box class O (FoxO), which negatively regulates cellular growth through transcriptional effects on downstream targets, including the translational repressor 4E-binding protein (4EBP, Thor) [39, 40]. In well-fed animals, in which insulin signaling and thus Akt are active, phosphorylated FoxO is excluded from the nucleus, thereby allowing growth to proceed, whereas under nutrient-restricted conditions, deactivation of Akt allows FoxO to enter the nucleus and act on its target genes, including *4EBP*, to suppress cell growth. Thus, the insulin/TOR pathway promotes nutrition-dependent growth partly through the inactivation of FoxO. Akt also mediates crosstalk with the TOR pathway through inhibition of  the Tuberous sclerosis complex 1 and 2 (Tsc1/2) proteins, which are negative regulators of TOR signaling. Therefore, TOR signaling senses internal nutritional status by two routes: via its diverse cell-autonomous nutrient-sensing mechanisms and through inputs from the insulin pathway via Akt [41, 42]. Although TOR has been known mainly for sensing free amino acids, recent work has shown that TOR activity is dependent on internal oxygen concentration as well [27, 43], indicating that TOR integrates both amino-acid and oxygen sensing to regulate cell growth in adaptation to changing environmental conditions. When TOR is active, it phosphorylates 4EBP, suppressing its inhibitory activity, which results in enhanced binding of mRNAs to ribosomes and thus in increased translation [44]. TOR signaling also promotes translation through the phosphorylation of ribosomal protein S6, mediated by S6 kinase (S6K), to enhance ribosomal activity [44].

Although the insulin/TOR signaling pathway directly regulates cellular and systemic growth rates, this pathway also controls the duration of the growth period by affecting ecdysone biosynthesis in the PG, which determines the onset of metamorphosis. Activating insulin/TOR signaling in the PG upregulates the expression of the Halloween genes *phantom* (*phm*) and *disembodied* (*dib*), which mediate ecdysone biosynthesis, leading to increased ecdysone production and thus to accelerated metamorphosis [9, 22, 45, 46]. Increased ecdysone signaling under these conditions results in the development of small adults not only due to the shortening of the larval growth period but also due to reduced growth rate, since ecdysone negatively regulates systemic growth. On the other hand, downregulation of the insulin/TOR pathway in the PG delays pupariation (the onset of metamorphosis), thereby increasing the growth period, which leads to overgrowth. Furthermore, overexpression of DILPs in the IPCs results in similar upregulation of *phm* and *dib* [47], indicating that ecdysone-mediated development can also be considered to be nutrition-dependent through the insulin pathway.

The TOR signaling pathway itself regulates the production of DILPs in *Drosophila* in response to amino-acid intake. Amino-acid sensing in the fat body via the TOR pathway controls DILP synthesis and secretion in the IPCs via inter-organ signaling [25, 48]. Recent studies have shown that a number of humoral factors are secreted from the fat body in an amino-acid-sensitive, TOR-dependent manner to regulate DILP expression in and secretion from the IPCs in the brain (Fig. 1); these factors include Growth-blocking peptide 1 (GBP1) and GBP2 [49], Stunted [50], Eiger [51], and Female-specific independent of transformer (Fit) [52] (Table 1). In addition to these  amino-acid-sensitive signals, a few dietary- sugar- and lipid-sensitive fat-body factors such as the type-I cytokine Upd2 [21], the Activin-like ligand Dawdle [53], and the small peptide CCHamide-2 [54] also regulate DILP secretion from the IPCs (Table 1). Thus, the fat body regulates DILP secretion in response to a number of dietary macronutrients, thereby coupling growth to nutrient intake, which is an important adaptive growth response of the organism to environmental conditions. In addition to its role in nutrition sensing, the fat body is also the main sensor of internal oxygen levels, which allows organisms to adapt their growth to environmental oxygen conditions through the regulation of DILP secretion [27]. Similar to low-amino-acid conditions that reduce growth via down-regulation of TOR, tissue hypoxia induced either by low environmental oxygen levels or by undergrowth of the tracheal airways also slows larval growth and development. This adaptive response requires oxygen sensing via the transcription factor Hypoxia-inducible factor 1 alpha (HIF-1a) in the fat body [27]. Fat-body hypoxia disinhibits HIF-1a activity, which in turn leads to the release of one or more unidentified fat-derived humoral factors that act on the IPCs to inhibit DILP expression and secretion. This HIF-1a-dependent fat-body oxygen-sensing mechanism strongly inhibits systemic insulin-dependent growth in response to tissue-hypoxia conditions. These conditions, at the same time, increase fibroblast growth factor (FGF)-like signaling, promoting the growth of the tracheal airway system to permit greater oxygen delivery to tissues. This adaptive growth and metabolic response promotes survival under environmental conditions with low oxygen. Furthermore, DILP secretion is also regulated by temperature, through a neuronal circuit involving a group of larval cold-sensing neurons that sense temperature fluctuation [28]. These neurons directly synapse upon the IPCs to activate the synthesis and secretion of DILPs in a temperature-sensitive manner. Taken together, the IIS/TOR pathway thus integrates amino acids, sugars, lipids, tissue oxygen, and temperature to control growth in response to changes in environment cues.

**Table 1:** Factors that regulate the IPCs in the larva, the adult, or both (some not discussed in the review)

IPC-influencing factors	Larval data	Adult data
Autonomous sugar sensing	No: sensing occurs via Akh relay [139]	Yes [140]
Autonomous amino-acid sensing	Via Minidiscs leucine transporter [137]	No adult data
Akh	From CC [139]	No adult data
AstA	Source undefined; via AstA-R2 [78]	Source undefined; via AstA-R2 [78]
CCHa2	From gut [180]; from fat [54]	*CCHa2* null affects insulin expression in the pupa via unspecified route [180]
Dawdle	Source undefined; unknown route to IPC effects [53]	No adult data
DILPs	No larval data	From IPCs and fat body; via InR [133, 149]
Eiger	From fat body; via Grindelwald receptor [51]	No adult IPC data
FIT	Not present in larvae [52]	From fat body; affects IPCs through unknown route [52]
GBPs	From fat body; via intermediating EGFR-expressing neurons [49, 179]	No adult data
Hugin	Neuronal source [272]	No adult data
Leucokinin	No larval data	Neuronal source [185]
Limostatin	No larval data	From CC [184]
PDF	No larval data	From clock neurons [188, 189]
sNPF	Reports differ: appears to operate in larvae [220]; appears not to operate in larvae [221]	From sugar-sensitive upstream neurons activates IPCs via sNPF-R [224]; from clock neurons [188]; also [219–221, 223]
Stunted	From fat body; via Methuselah receptor [50]	No adult data
Tachykinin	TkR99D likely present [206]	Source undefined but brain suggested; via dTkr (TkR99D) [206]
Upd2	From fat body; via Domeless receptor in presynaptic GABAergic neurons [21]	From fat body; via Domeless receptor in presynaptic GABAergic neurons [21]
Unknown ligand	Ligand and source unknown; via AdipoR [289]	Ligand and source unknown; via AdipoR [289]
Unknown hypoxia-induced ligand	From fat body [27]	No adult data
Dopamine	No larval data	Via DopR1 [290]
Ecdysone	E → 20E in fat body; EcR in IPCs [86]	No adult data
GABA	GABA-B-R2 present in IPCs but no further findings [291]	[291]
Serotonin	*5-HT1A-GAL4* is not expressed in feeding third-instar larval IPCs [292]	Via 5-HT1A [290, 292]
Lipid particles	Via accumulation of lipid particles on neurons presynaptic to IPCs [293]	No adult data
Taotie neurons	No larval data	Neurons upstream of IPCs [294]
Temperature	Cold-sensing neurons presynaptic to IPCs [28]	No adult data

### Integrating photoperiod, organ growth status, and nutritional information through PTTH signaling

It is critical for animals such as insects to synchronize their developmental transitions to daily environmental cycles, and therefore  the systems controlling developmental timing are under photoperiod control. Furthermore, developing organisms also need enough time to complete the growth of their organs, as well as the adaptive plasticity to adjust their growth to compensate for impaired tissue growth or injury, to ensure developmental stability. These adaptive responses, which maximize survival and reproductive success, require the integration of photoperiod and organ-growth status with developmental programs. Photoperiodic inputs and tissue-damage signals are integrated by the PTTHn, two pairs of neurosecretory cells in the larval brain that produce PTTH and directly innervate the PG [55]. PTTH controls developmental timing through its effects on the PG, where it activates its receptor tyrosine kinase Torso, leading to the pulse of ecdysone production that initiates metamorphosis [56]. Activated Torso stimulates the phosphorylation of extracellular signal-regulated kinase (ERK) through the canonical MAPK pathway including Ras, Raf, and MEK. Ablation of the PTTHn, loss of PTTH in these cells, or loss of Torso or ERK in the PG delays larval development in *Drosophila* due to delayed production of ecdysone in the PG. Thus, PTTH is an important neuropeptide that regulates growth duration in *Drosophila* [55, 56]. The PG undergoes apoptosis during metamorphosis; in adults, ecdysone has non-developmental functions and is thought to be produced in the gonads [57–61].

The PTTHn integrate developmental and environmental cues to adjust the length of the growth period during larval development by changing the timing of PTTH secretion. For instance, photoperiod strongly affects PTTH secretion in a broad range of insect species, although *Drosophila* shows weak responses compared to other insects [62, 63]. During larval development, the PTTHn are regulated by neurons producing the neuropeptide Pigment-dispersing factor (PDF), which are known to be associated with the circadian clock and to receive input from photoreceptors in Bolwig’s organ, the larval light-sensing tissue [55, 64]. Furthermore, beyond controlling the developmental growth period by determining the timing of metamorphosis, PTTH also coordinates larval behavior with this developmental transition to maximize survival. PTTH acts via Torso on two light sensors, the Bolwig’s organ and the peripheral class-IV dendritic arborization neurons, in developing *Drosophila* larvae to control light-avoidance behavior, ensuring that the animals stay in dark environments that minimize the risk of desiccation and predation [63]. The PTTH neurons themselves may be regulated by transitions in light intensity, forming a feedback loop between development, environment, and the nervous system [65].

When insect larvae face abnormality in tissue development, such as injury, accidental asymmetric growth of a paired organ, tissue overgrowth, or tumorigenesis, they slow their development to allow time for healing or regeneration [66–68]. In response to abnormal growth, the tissue primordia that give rise to adult appendages—the imaginal discs—secrete DILP8 [69, 70], which delays metamorphosis by changing the timing of ecdysone peaks. DILP8 secreted by abnormally growing organs is sensed by the receptor Lgr3 in a pair of neurons that synapse upon the PTTHn [71–73], suggesting that abnormal organ growth delays developmental timing primarily by affecting the timing of PTTH secretion. DILP8 also affects the growth-controlling DILPs via contact between Lgr3 neurons and the IPCs [73], suggesting that it coordinates growth (through regulation of DILPs) and maturation (through regulation of PTTH).

Developmental coordination between growth and maturation is also mediated by the neuropeptide Allatostatin A (AstA) and its receptor AstA receptor 1 (AstA-R1), which regulate developmental timing by controlling PTTH and insulin signaling [74, commentary in 75]. RNAi-mediated knockdown of *AstA-R1* in the PTTH-producing cells impairs PTTH secretion. Moreover, AstA-R1 also stimulates DILP secretion from the IPCs [74]. Interestingly, AstA and AstA-R1 are homologous to human kisspeptin (KISS) and its receptor GPR54 [76], which are known to be required for human puberty through their control of gonadotropin-releasing hormone (GnRH) secretion from the brain, which initiates maturation by inducing sex-steroid production [77]. This suggests that the neuroendocrine architecture that controls the initiation of maturation has been evolutionarily conserved and that this system in *Drosophila* coordinates developmental growth with the juvenile-to-adult transition to achieve an appropriate size under different environmental conditions to maximize adult fitness. AstA is regulated by nutrition, at least in adults [78], suggesting that in addition to photoperiod and organ-growth status, nutrition may modulate PTTH secretion. This is in line with a recent report showing that PTTH secretion is regulated by amino-acid levels [79].

Furthermore, studies in lepidopterans have indicated that PTTH secretion is gated not only by the photoperiod but also by JH, which represses ecdysone biosynthesis and metamorphic development [30]. One of the functions of JH is to change the duration of the growth period by modulating the timing of PTTH and ecdysone release [62]. Although it is not clear whether JH regulates PTTH in *Drosophila*, removing the corpora allata (CA), which comprises the JH-producing cells, induces developmental delay [80], suggesting a potential interaction with ecdysone production. This may occur through PTTH signaling, as seen in other species. The transcription factor Krüppel homolog 1 (Kr-h1), which mediates JH signaling, has been shown to attenuate ecdysone biosynthesis in the PG by directly inhibiting the expression of the “Halloween” biosynthetic enzymes [81]. Since the mechanism by which JH might affect PTTH is unknown in *Drosophila*, future studies should determine whether JH signaling through Kr-h1 regulates PTTH.

Taken together, recent advances have shown that the PTTHn integrate several intrinsic and extrinsic cues to modulate the timing of steroid-hormone production and secretion, and thus developmental maturation, by modulating the timing of PTTH secretion. PTTH, therefore, seems to be the key factor in the adaptive plasticity that allows animals to adjust development to variable environmental conditions. To achieve such flexibility, the neuroendocrine network controlling PTTH, the principal regulator of maturation in *Drosophila*, likely integrates a wide range of inputs to control PTTH secretion. Understanding how internal and external cues are integrated via PTTH signaling will be a key direction for future research.

### The larval prothoracic gland is a center for the integration of signals

PTTH is the primary factor stimulating ecdysone production in the PG, according to the classical model of the insect neuroendocrine system. However, it has become evident in recent years that the PG itself functions as a decision-making center that integrates a broad array of cues. During *Drosophila* larval stages, the PG is part of the major endocrine organ called the ring gland that also comprises the JH-producing CA and the Akh-producing cells (APCs) of the corpora cardiaca (CC). Ecdysone is synthesized from sterols in the PG in a series of reactions mediated by enzymes encoded by the so-called Halloween genes [82, 83]. Ecdysone produced and released into circulation by the PG is converted to a more biologically potent form, 20-hydroxyecdysone (20E; ecdysone is used here interchangeably with 20E for simplicity), by another Halloween enzyme, Shade, in peripheral tissues such as the fat body [59, 84, 85]. Interestingly, Shade-mediated 20E production by the fat body is nutrient-dependent, and peripherally produced 20E itself regulates the IPCs, indicating multidirectional linkage between nutrition and steroid-hormone activation in peripheral tissues [86].

Ecdysone binds to a heterodimeric nuclear hormone-receptor complex, consisting of the ecdysone receptor (EcR) and its partner Ultraspiracle (Usp) [87–89], that regulates transcriptional responses to ecdysone [90]. In response to a wide range of signals, the PG generates a pulse of ecdysone that induces wandering behavior, which terminates feeding in the final larval instar and ultimately leads to pupariation. Therefore, ecdysone is considered to be a primary factor for ending the juvenile growth period, thereby limiting growth duration and determining adult size [13, 31, 91]. In the third and final larval instar, three small ecdysone pulses followed by a large pulse are believed to drive developmental progression [92]. The third small pulse is associated with the cessation of feeding and the onset of wandering behavior, in which animals leave the food to find appropriate pupariation sites [13, 92]. Although PTTH plays a key role in stimulating ecdysone production in the PG, this tissue itself also senses organismal nutritional status. Insulin and TOR signaling in the PG works upstream of ecdysone production and adjusts it to match nutritional status [9, 22, 46]. Insulin appears to govern ecdysone biosynthesis through effects on the Warts-Yorkie-*bantam* pathway, which regulates delivery of the steroid precursor cholesterol for ecdysone biosynthesis through an autophagosomal cholesterol-trafficking mechanism [10, 93]. Autophagy is a conserved mechanism for the degradation and recycling of intracellular components that is involved in cellular adaptation to starvation; autophagy-dependent ecdysone regulation controls basal ecdysone levels, which regulates the growth rate, rather than the ecdysone peak that determines the growth period by triggering the onset of metamorphosis. In the PG, this nutrient-dependent mechanism allows animals to adapt organismal growth to nutritional conditions through regulation of ecdysone synthesis.

In addition to brain-derived signals, the PG also receives information from other tissues such as the gut and imaginal discs. In developing *Drosophila* larvae, the gut senses nutrient availability and produces a circulating lipoprotein-associated form of Hedgehog (lipo-Hh). Circulating lipo-Hh directly acts on the PG to regulate ecdysone biosynthesis [94]. In addition, a subset of serotonergic neurons also affect ecdysone production in a nutrition-dependent manner [95]. Larvae raised on a yeast-poor diet with low amino-acid content grow more slowly; under this condition, certain serotonergic neurons sparsely innervate the PG, whereas these neurons arborize extensively onto the PG when animals grow rapidly on a yeast-rich diet. Moreover, blocking serotonin signaling from these neurons delays larval development, suggesting that they regulate ecdysone production in response to internal nutritional conditions [95]. Furthermore, TGF-β signaling via the ligands Activin and Decapentaplegic (Dpp) appears to regulate ecdysone production in the PG. Blocking the TGF-β/Activin signaling pathway in this tissue results in animals that fail to initiate metamorphosis and thus persist as feeding, growing larvae, eventually attaining a giant size [96], a phenotype typically associated with failure of ecdysone production. Consistent with such a failure, reducing TGF-β/Activin signaling in the PG downregulates expression of both the PTTH receptor Torso and the DILP receptor InR, suggesting that TGF-β/Activin signaling in the PG is necessary to induce its competence to respond to PTTH and insulin signals [96]. Thus, TGF-β/Activin signaling appears to be necessary to ensure that both developmental and nutritional prerequisites are met before metamorphosis is triggered, although the nature and source(s) of the TGF-β ligand(s) that act upon the PG remains an open question [97]. Conversely, Dpp derived mainly from imaginal discs acts on the PG through TGF-β/BMP pathway to repress ecdysone biosynthesis, at least in part by interacting with insulin/Warts/*bantam* signaling [98]. Dpp is more commonly known as a disc morphogen, similar to Hh; its signaling from the discs to the PG suggests that it might represent an additional mechanism by which the endocrine system assesses the patterning and growth status of developing organs to make the irreversible "go/no-go" maturation decision.

Circadian rhythms also govern insect development; this has been generally reviewed elsewhere [99]. Of particular interest here is the suggestion that PG physiology is governed in a circadian fashion. As discussed above, the PTTHn receive circadian input; however, the PG also possesses an endogenous peripheral clock that drives cyclical changes in gene expression, including that of *InR*, which is downregulated at subjective “night,” when feeding is reduced and insulin levels fall [100]; in the proposed model, circadian downregulation of insulin signaling potentiates PG-activating Torso signaling [100]. Although the specifics of this model are somewhat surprising, one may speculate that, in general, matching of the rhythms of (1) feeding behaviors and insulin, (2) light-induced rhythmicity of PTTH, and (3) the PG-intrinsic clock optimizes the timing of ecdysone production.

### Neuroendocrine signaling hubs integrate developmental and environmental cues

Blocking either PTTH/Torso signaling [55, 56] or DILP/InR/PI3K signaling [9, 22, 47] alone in the PG induces a delay in pupariation, whereas simultaneously blocking both signaling routes into the PG results in a failure to pupariate due to the lack of ecdysone production [96]. These observations suggest that PTTH and DILPs are the major PG-extrinsic signals that regulate ecdysone production. Since the PTTHn and IPCs are sensitive to a number of different intrinsic and extrinsic stimuli, and the PG itself also senses changes in environmental and internal cues, we propose that this neuroendocrine network between the PTTHn, IPCs, and the PG acts as a cue-integrating hub for environmental and developmental signals (Fig. 1). Because insulin and ecdysone are the key regulators of growth rate and duration, organismal adaptation of growth and development to environmental conditions is mediated by the integration of signals through this neuroendocrine hub.

Under favorable food and oxygen conditions, active insulin signaling induces rapid growth and at the same time promotes ecdysone production, which accelerates metamorphosis. In contrast, when larvae are exposed to unfavorable conditions, reduced insulin signaling slows ecdysone production, prolonging the growth period by delaying metamorphosis (Fig. 1). In addition to nutritional and oxygen inputs, a developmental checkpoint for tissue growth and injury is processed by the PTTHn and IPCs. Growing and damaged discs release DILP8, a signal that regulates insulin signaling and suppresses PTTH secretion, which extends the growth period by delaying metamorphosis, mediating plasticity that promotes developmental stability. Furthermore, photoperiodic input is mediated by PTTH signaling, while temperature is relayed to the neuroendocrine system by the IPCs, which receive inputs from cold-sensing neurons. Thus, temperature can affect ecdysone indirectly via DILP-mediated regulation of synthesis in the PG of *Drosophila*. Oxygen and temperature may also be integrated by the PG itself, as suggested from studies in other insects [101, 102]. Interestingly, ecdysone regulates growth negatively in larval tissues in *Drosophila* through a fat-body relay mechanism that inhibits systemic insulin signaling [9, 103]. Reducing ecdysone signaling specifically in the fat body results in an increased growth rate. In suboptimal nutritional conditions, relatively high ecdysone levels seem to suppress growth. Thus, both ecdysone and insulin fine-tune growth rate and duration to produce a species-specific adult body size in response to changes in environmental and internal conditions.

### Regulation of the growth period by a nutritional checkpoint

In insects, one of the most important environment-sensitive checkpoints that ensures an appropriate adult body size under different nutritional conditions is called “critical weight.” Before this checkpoint is satisfied, developmental progression is nutrition-dependent [30]. In contrast, when critical weight is reached, larvae become committed to undergoing metamorphosis into adults on a fixed schedule irrespective of further nutritional inputs. Thus, critical weight is a checkpoint-based mechanism that ensures that animals adjust their larval growth period to nutritional conditions, extending its duration under conditions of nutrient scarcity, in which critical weight is reached after prolonged feeding. However, this raises questions regarding the nature of the molecular mechanism by which *Drosophila* and other animals sense their own size and critical-weight attainment during development. *Drosophila* larvae appear to rely on nutritional status rather than actual body size, which seems to be similar to the mechanism that governs mammalian maturation [31, 104]. Insect metamorphosis is the key developmental event in the juvenile-to-adult transition in holometabolous insects, analogous to mammalian puberty. Both metamorphosis and puberty are ultimately orchestrated by steroid hormones, which are tightly regulated by the activation of a neuroendocrine signaling cascade, suggesting that the architecture of the system that triggers maturation is conserved.

The first clear description of the *Drosophila* nutritional checkpoint based on the relationship between nutritional input and the duration of the growth period was made almost a century ago [105]. Later, this developmental checkpoint was named “critical weight” based on observations in the lepidopteran *Manduca sexta* [30]. Critical weight generally occurs early in the final larval instar and triggers a cascade of events that ultimately initiates the terminal growth period, which is the period between critical-weight attainment and the onset of metamorphosis. Thus, while pre-critical-weight animals can extend their growth period under nutrient-poor conditions to compensate for slow growth, the post-critical-weight terminal growth period is largely fixed in duration and cannot be extended even by starvation. However, environmental factors do still govern the animal's growth rate during the terminal growth period, and thus adult size is largely determined by the conditions prevailing during this window.

Wild-type *Drosophila* larvae developing at 25 °C under normal atmospheric oxygen levels (~ 21%) reach critical weight 8–12 hours after the molt to the third and final instar [22, 55, 106–112], which coincides with a small nutrient-sensitive pulse of ecdysone [92, 113]. This rise in ecdysone is believed to result from nutrient-dependent insulin/TOR signaling in the PG and is thought to underlie the critical-weight transition in *Drosophila*, since pre-critical-weight larvae fed ecdysone pupariate without delay when starved [106]. Consistent with this notion, insulin signaling gradually increases in the PG when newly molted third-instar larvae feed continuously [106]. Furthermore, activating insulin/TOR signaling in the PG induces precocious critical-weight attainment, whereas reducing it delays this [9, 22, 45, 46, 106, 114]. One hypothesis proposes that this small nutrient-sensitive ecdysone peak is caused by increased insulin signaling [106]; another holds that nutrient-dependent TOR-mediated progression of endocycles of chromosomal replication in the cells of the PG leads to an irreversible activation of ecdysone biosynthesis that triggers the critical-weight transition [110, 115]. Notably, these hypotheses are not mutually exclusive, and perhaps rising insulin signaling is able to activate an ecdysone pulse only after enough chromosomal duplication has occurred to induce a transcriptional state that commits the PG to synthesize ecdysone. In any case, taken together, these observations suggest that critical weight depends on insulin/TOR signaling in the PG that is correlated with the nutritional condition of the animal, rather than its body size *per se*. In addition to nutrients, other intrinsic and extrinsic factors also affect critical weight. In hypoxic conditions, *Drosophila* larvae reach critical weight at a smaller size, which results in reduced adult size [116]. Temperature also affects this developmental checkpoint: at lower temperatures, animals including *Drosophila* reach larger adult sizes at least partially because larvae tend to reach critical weight later, at a larger size [112]. Furthermore, sex-dependent size differences can also be explained partially through effects on critical weight [109].

Once animals reach critical weight, they commit to releasing PTTH, which triggers the neuroendocrine signaling cascade leading to the maturation-inducing ecdysone pulse that initiates metamorphosis. Since PTTH secretion from the PTTHn is an outcome of the critical-weight transition, modulation of the PTTH receptor Torso in the PG or ablation of the PTTH-producing cells induces phenotypes similar those observed in animals with altered insulin signaling in the PG [55, 56]. In this scenario, PTTH is required for the animal to respond to critical weight, which depends on an insulin/TOR-mediated rise in the ecdysone production in the PG. Alternatively, signaling through insulin/TOR and PTTH collectively is responsible for generating the first small ecdysone peak that triggers the critical-weight transition. Animals lacking PTTH reach critical weight later at a larger size, suggesting that PTTH signaling is important in setting critical weight [111]. Furthermore, *Ptth* mutants are delayed in the terminal growth period, but eventually do pupariate and develop into adults, suggesting that other signals are sufficient to drive ecdysone production in the PG. During the prolonged feeding period of animals lacking PTTH signaling, the additional accumulation of nutrients and thus increased adiposity may eventually induce ecdysone signaling through increased insulin signaling. Thus, the PTTH, insulin, and TOR pathways are key to integrating environmental cues and internal nutritional status to coordinate growth and developmental transitions.

This evidence suggests that nutritional factors and nutrient sensing, rather than organismal size, are used to assess the attainment of critical weight. The *Drosophila* larval fat body is the primary nutrient-storage organ, and it also acts as a central nutrition sensor. In response to nutrient intake, the fat body secretes a number of insulin-regulatory factors, which couple growth to nutritional conditions by remote control of DILP secretion from the IPCs (Table 1). During development, the fat body senses adipose storage of nutrients and relays that information to control insulin signaling, which promotes the ecdysone production that triggers the critical-weight transition. In a similar phenomenon observed in humans, body weight strongly correlates with the timing of menarche, leading initially to the use of the term “critical weight” for humans [117–119]. However, human “critical weight” appears to arise from effects due more specifically to adiposity, rather than overall body size. Obese children tend to undergo puberty earlier than non-obese children of similar height, whereas malnourished children who lack body fat exhibit delayed puberty [120]. In this model, the neuroendocrine pathways controlling maturation onset in humans thus likely receive input from hormones produced by adipose tissues. Interestingly, in mammals, including humans, the adipokine leptin regulates pubertal maturation [121]. Leptin concentrations in the bloodstream reflect adiposity, and leptin deficiency causes a failure to undergo puberty. In *Drosophila*, the functional analog of leptin is the adipokine Upd2; this factor is released from the fat body in a nutrient-dependent manner and from the musculature in response to daily activity cycles, and it regulates insulin secretion from the IPCs and Akh release from the APCs [21, 122]. Based on these similarities, one might speculate that in the *Drosophila* larva, the adipose tissue releases one or more humoral factors in response to stored nutrient levels and, further, that these signals act via the IPCs to promote DILP release onto the PG, signaling that larvae have accumulated sufficient nutrients to undergo successful metamorphosis and to maximize fitness in adulthood.

## Metabolic and behavioral adaptation to changing environments

**Fig. 2:**
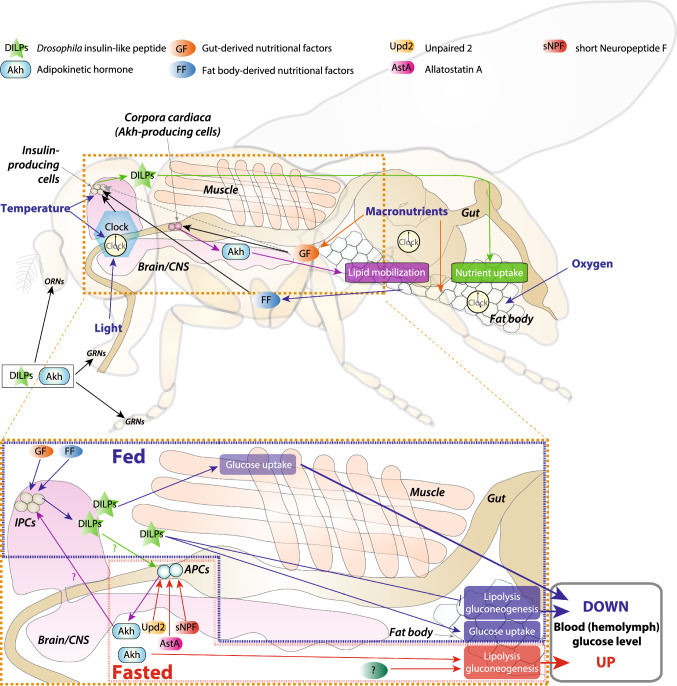
Metabolism and behavior are regulated via the integration of environmental and internal cues through inter-organ communications in *Drosophila* adults. The top panel shows adult organs and the diffusible factors that link them to control metabolism and feeding behaviors. Circadian clocks are located within the brain as well as in peripheral tissues and regulate tissue physiology. Gustatory and olfactory receptor neurons (GRNs and ORNs) are regulated by DILP and Akh signaling (as well as many other factors) and influence feeding behavior. The bottom panel schematizes adult organs and interactions that govern the level of circulating sugars

Both during and after their development, organisms must adapt their metabolism to maintain energetic homeostasis under the changing current environment as well as to anticipate near- and distant-future conditions. In animals, these metabolic adaptations require a balance between energy consumption and utilization through regulation of nutrient intake, storage, and expenditure. This metabolic flexibility relies on endocrine signaling networks that control tissue-specific adjustment of carbohydrate, amino-acid, and lipid metabolism, as well as signals that regulates locomotion, feeding, and reproduction, all of which have a large impact on energy balance (Fig. 2). The tight linkage between growth and metabolic control in *Drosophila* means that many of the systems that regulate larval growth and development also play a role in adult metabolic control.

In both mammals and insects, well-fed conditions lead to an increase in circulating sugar levels, which induces the release of insulin or insulin-like peptides that promote cellular energy uptake either for immediate use or for storage as a buffer against future scarcity. Flies, like mammals, store excess energy in the form of tri- and diacylglycerides (TAGs and DAGs), primarily in the fat body (functionally analogous to mammalian liver and adipose tissues [2, 123]), as well as the branched glucose polymer glycogen, largely in the larval and adult musculature [124, 125], fat body [125–127], and nervous system [128]. Both groups of animals also produce a hormone that counters the actions of insulin-like signaling when circulating sugar levels drop because of physical activity (high depletion) or starvation (insufficient supply) by promoting the breakdown of stored energy into circulating species. Glucagon plays this role in mammals; in insects, this function is primarily performed by Akh.

### Drosophila insulin-like peptides (DILPs) govern cellular energy uptake and storage

In mammals, insulin is secreted by the pancreatic β cells in response to high blood sugar levels and promotes the cellular uptake and utilization or storage of glucose to prevent hyperglycemia. This system is evolutionarily ancient, and an orthologous system exists in insects. In the fly, DILPs (introduced above) regulate the uptake of metabolic species, including sugars. Within the brain, the larval IPCs—which are genetically homologous to the mammalian β cells [12, 129, 130]—persist through metamorphosis into the adult and produce a context-dependent mixture of DILP1, DILP2, DILP3, and DILP5, as well as the cholecystokinin orthologue Drosulfakinin (Dsk) [131]. In addition, larval Dh44-producing cells are also recruited into an insulin-producing role in the adult, secreting DILP2 (in addition to Dh44, but not DILP3 or -5) onto or around the foregut/crop [132]. DILP6, produced in the fat body of the non-feeding pupal stage to promote metamorphic growth [35, 36], is also upregulated in the larval and adult fat body during starvation [133].

Cells that produce metabolism-regulating hormones such as DILPs and Akh must be able to sense the animal’s nutritional state, either cell-autonomously or via other signals, in order to respond with appropriate hormonal cocktails (see Fig. 2 and Table 1). Mammalian insulin-producing pancreatic β cells respond directly to blood glucose. Imported glucose leads to ATP production, increasing the ratio of ATP to ADP, which results in the closure of ATP-sensitive K^+^ channels and depolarization of the cells. In turn this leads to opening of voltage-gated Ca^2+^ channels and endocrine secretion of insulin. Similarly, mammalian glucagon-producing pancreatic α cells are directly regulated by sugars via ATP as well and release glucagon under low sugar levels, although some mysteries remain regarding the precise mechanisms involved [134–136]. In *Drosophila* larvae*,* insulin secretion is tightly linked to amino-acid intake during development [25, 48], since DILPs are the major growth factors. Larval IPCs sense the amino acid leucine via the protein Minidiscs and upregulate DILP2 and DILP5 in response to higher leucine availability [137]. Although sugar also affects larval DILP signaling, the larval IPCs do not appear to be competent to respond directly to sugar levels, indicating that they are not directly regulated by intracellular sugar sensing [138]; rather, a relay via Akh appears to regulate IPC sugar responses [139]. Isolated adult IPCs, however, do appear to be directly sugar-responsive in their electrical activity, suggesting that the IPCs of the adult fly are regulated via a glucose-sensing mechanism similar to that of mammalian insulin-producing β cells [140].

Each of the DILPs is under independent transcriptional and secretory regulation. Their relative expression varies over developmental time during larval life [141]. Furthermore, in the larva and the adult, each DILP-encoding gene is responsive to different nutritional cues [11, 139, 142], enabling the animal to adapt its metabolism to a broad variety of nutritional combinations. Indeed, within the nutritional space encountered by *Drosophila* in the wild (i.e., the range nutrients associated with rotting fruits), adult *Dilp2* expression appears to be upregulated by high ratios of carbohydrate to protein in the diet, whereas in contrast, *Dilp3* shows an irregular expression profile in adults, with a peak of expression on a diet of roughly 8% sugar and 1% protein [142], which suggestively approximates the composition of natural fruits. Adult transcription of *Dilp5* appears to increase with the overall calorie level of the diet [142], whereas adult *Dilp6* expression does not vary much with food composition in fed conditions [142] and appears to be influenced primarily by starvation [133].

Whereas the growth and metabolic functions of mammalian insulin-like factors are divided into parallel pathways, with insulin and its receptor governing metabolism and the IGFs and their cognate receptors (IGFRs) controlling growth, the fly expresses only a single insulin receptor, which responds to multiple DILPs and regulates both growth and metabolism. Thus, to be able to induce alternative downstream responses, the DILPs exhibit varying biochemistry. These peptides are varied in sequence and structure (e.g., DILP2, 3, and 5 are likely processed by cleavage into A and B chains, with the removal of the intervening “C peptide,” whereas DILP6, like mammalian IGFs, is likely not cleaved [32, 35]). These differences allow them to bind with different kinetics to the insulin receptor and thereby to bring about alternative intracellular responses [143]. In addition, several hemolymph proteins—*Drosophila* Acid-labile subunit (dALS), Ecdysone-inducible gene L2 (ImpL2), and Secreted decoy of InR (Sdr)—differentially bind circulating DILPs and modulate their interaction with InR, thus further functionally differentiating the DILPs from one another. dALS appears to be required for efficacious signaling of DILP2 and DILP5, but it does not bind DILP3 [144]. ImpL2 is released during poor nutritional conditions and sequesters circulating DILPs to block their activity [145]—most strongly interacting in ex-vivo pulldown assays with DILPs 1, 2, 5, and 6 and more weakly with DILPs 3 and 4 [146]—while at the same time promoting local DILP2 actions at specific anatomical sites [147, 148]. In contrast, Sdr most strongly binds DILP3 in pull-down assays, but it also can interact with DILPs 1, 2, and 7, and to a lesser degree with DILPs 5 and 6 [146]. Many of these factors modulating circulating DILPs have mainly been studied during development, but they likely play similar roles in adults. Thus, even though all DILPs act through the same receptor, the DILP system offers broad functional flexibility to allow different nutritional stimuli to induce a range of intracellular adaptive responses in the face of a range of dietary inputs. Furthermore, complex feedback-regulatory relationships control *Dilp* expression; DILP2, DILP5, and DILP6 act as negative regulators of DILP-gene expression, while DILP3 feeds back positively via either autocrine action or intermediate signals [133, 149]. This dynamic transcriptional interplay further fine-tunes expression of *DILP* genes to produce the complex mixtures necessary to homeostatically regulate the internal metabolism of the fly.

In addition to the DILPs, the IPCs also produce the peptide hormone Drosulfakinin (Dsk), which is an orthologue of mammalian cholecystokinin [131, 150]. This peptide has been studied in a variety of insects and has a range of functions in signaling satiety and regulating food intake. *Dsk* transcription is reduced upon starvation, and *Dsk*-depleted animals consume significantly more food, whereas Dsk peptide injection conversely reduces nutrient ingestion [131, 151–153]. Moreover, Dsk appears to reduce olfactory sensitivity to attractive odors in larvae [154] and to inhibit the consumption of unpalatable food in adults [131], consistent with a role in not only regulating food intake, but also in the neuronal processing that underlies food choice. As demonstrated for human cholecystokinin [155], Sulfakinin-family proteins also regulate critical aspects of gut physiology in a variety of insect systems: in the locust, Sulfakinin injection reduces secretion of digestive enzymes [156], while there is evidence that it may act as a regulator of gut/crop contractions in adult *Periplaneta* and larval *Drosophila* [157]. Taken together, this pleiotropic peptide thus appears to regulate many aspects of feeding behavior, making Dsk a key player in the regulation of metabolic stability across a range of animal systems.

### Adipokinetic hormone (Akh) governs the mobilization of energy reserves

Maintaining biological functions under negative energy balance depends on the release of a hormone that instructs tissues to mobilize stored energy reserves in order to make sugars and lipids available to peripheral tissues. Metabolic homeostasis in complex animals is thus reliant on constant communication between nutrient-storing and nutrient-consuming tissues to offset potential deleterious fluctuations in circulating energy levels during periods of energy stress. In insects, the best-studied nutrient-mobilizing hormone is Akh, which induces glycemia-increasing effects similar to those of mammalian glucagon (Fig. 2). It is worth noting that although the Akh and its receptor AkhR are functionally analogous with glucagon and its receptor, these two systems are not closely evolutionarily related. Whereas glucagon achieves its glycemic effect by inducing glycogenolysis, with possible effects on lipids whose nature and relevance are controversial [158], Akh in *Drosophila* appears to act primarily as a lipolysis-inducing factor. Although loss of Akh function in larvae does not increase fat stores under normal conditions [159, 160], larval Akh overexpression does reduce fat stores [160]; disruption of Akh signaling in adults partially blocks lipid mobilization under starvation [161] and results in larger fat stores [159, 162]. Reports of Akh effect on glycogen, however, vary. Most studies for which glycogen levels are reported have found no effect of Akh-signaling disruption on larval or adult glycogen levels [126, 159, 161]; however, another report finds that *AkhR* loss results in slightly increased adult glycogen levels and that *AkhR* overexpression (driven by *AkhR-GAL4*) reduces adult glycogen levels, both effects becoming more pronounced after starvation [162]. Akh-independent mechanisms of lipid and glycogen mobilization also exist and are discussed below.

In both larval and adult *Drosophila*, prepro-Akh is expressed by the neuroendocrine APCs of the CC [163]. The prepropeptide is enzymatically processed [164, 165] into the N-terminally phosphorylated, C-terminally amidated Akh octapeptide and an Akh precursor-related peptide (APRP). Akh peptide has been mass-spectrometrically identified in adult [159, 164–166] and larval [164, 167] CC-associated tissues, and APRP has recently been observed in adult tissues [159], thus confirming prepropeptide processing and production of active peptide.

The release of the bioactive peptide into the hemolymph from the APCs appears to be induced cell-autonomously by low hemolymph sugar (trehalose) levels, although exogenous factors, discussed below, impose additional control (Table 2). Extracellular trehalose levels affect APC cytoplasmic glucose levels, which in turn govern the ATP-producing activity of the mitochondria; low hemolymph sugar thus leads to reduced ATP production and a greater ratio of AMP to ATP. This ratio is detected by the actions of the AMP-activated protein kinase (AMPK) complex, which as in mammals integrates internal energy cues to modulate APC excitability and Akh release [168]. ATP-dependent membrane-associated K^+^_ATP_ channels also regulate cell excitability; these channels act as cellular AMP/ATP sensors that couple rapid decreases in sugar levels to the activation of voltage-sensitive Ca^2+^ channels and thus to hormone release [138]. These intracellular mechanisms show remarkable functional analogy to mammalian glucagon release from pancreatic islet α cells [169].

**Table 2:** Factors that regulate the APCs in the larva, the adult, or both

APC-influencing factors	Larval data	Adult data
Autonomous sugar sensing	Via K_ATP_ channels [138] and AMPK [168]; also [139]	Via AMPK [168]
Akh/AkhR feedback	No larval data	Negative feedback (at least indirect) [159]
AstA	Source unidentified; via AstA-R2 [78]	Source unidentified; via AstA-R2 [78]
Bursicon-Alpha	No larval data	From gut; inhibits CC via unspecified neuronal relay [200]
sNPF	No larval data	From sugar-sensing neurons presynaptic to CC; inhibits via sNPF-R [224]
Upd2	No larval data	From muscle to CC; via Domeless [122]

Interestingly, Akh release is also reported to be induced by hypertrehalosemia in *Drosophila* larvae [139], which was further supported by a recent study showing that chronic exposure to a high-sugar diet induces a prominent Akh-dependent response in the fat body [170]. These results suggest that Akh secretion is biphasically regulated by both low- and high-hemolymph trehalose concentrations, which may be interpreted as a mechanism necessary to support the high energy demands during rapid larval growth as well as the requirement to maintain normoglycemia during the wandering and pupal stages when feeding has ceased. Intriguingly, similar paradoxical glucagon stimulation has been described from isolated mouse pancreatic islets [171], just as humans with severe diabetes often show pronounced hyperglucagonemia [172], indicating that biphasic hormone release may be an evolutionarily ancient mechanism conserved since the divergence of insects and mammals. Whether this biphasic release also exists in adult *Drosophila*—a stage with fundamentally different physiological requirements—is unknown and represents an exciting question for the future.

The *Drosophila* genome encodes a single Akh receptor (*AkhR*), which is strongly expressed in fat-body cells, consistent with the energy-mobilizing roles of the Akh signaling system [173, 174]. Ablation of the cells of the CC [138, 160, 164, 175], prevention of the proteolytic processing of prepro-Akh [164], precisely targeted disruption of the genomic region encoding the processed Akh peptide [159, 176], and manipulation of AkhR [139, 161, 162] have been used to probe the Akh signaling pathway. The pathway does not appear to be necessary for larval survival or growth on normal diets, although *AkhR* mutants develop quite slowly on low-protein (low-yeast) food, likely due to effects mediated by effects on DILP3 [139]. Pathway loss by any means generally leads to reduced circulating sugar levels in larvae and adults, with little or no effect on larval lipid stores, at least in feeding larvae; however, starvation induces much stronger reduction of circulating sugars in larvae lacking CC cells than in controls, suggesting that the Akh deficient animals are unable to mobilize stores such as lipids [138]. Inactivation of the Akh pathway in adults, however, induces obvious phenotypes: adults with impaired Akh signaling exhibit reduced (but not eliminated) lipid mobilization, leading to increased lipid stores. Akh/AkhR phenotypes are especially marked under starvation—the reduction of the lipid mobilization rate allows lipid stores to be maintained longer, prolonging survival under starvation, and animals eventually succumb with substantial remaining fat stores [159–162, 175, 176].

### The IPCs and APCs are also regulated by exogenous factors

In metazoans, different aspects of the work of life are distributed among discrete specialized organs. Each organ has direct access to only a part of the information available to and within the whole animal, and therefore, to maintain homeostasis, organs coordinate their activities through the interchange of inter-organ signals as well as neuronal networks. In particular, the gut, fat, and nervous system release many neuropeptides and hormonal signals in response to cues that they are specialized to perceive. The gut, as the first organ to encounter ingested nutrients, is the source of many “phasic” factors that likely reflect recent nutritional intake, whereas the fat, as a central organ of metabolite storage and processing, produces “tonic” signals reflecting internal metabolite levels. The nervous system serves as an integrator and processor of multiple streams of hormonal, sensory, and behavioral information. The IPCs make up one key hub for the relay and integration of many neuronal and hormonal inputs from different tissues (Table 1); these modulate the expression and release of DILPs and Dsk. Several excellent comprehensive reviews of the influences that regulate DILP production and release have been published [15, 177, 178], and, therefore, only certain factors will be discussed in detail below. Likewise, although the hormonal regulation of APC activity has not been systematically investigated, some factors that govern Akh expression and release have been identified (below and Table 2).

### Signals that regulate the IPCs

The DILPs and Dsk are involved in a range of physiological and metabolic processes. To coordinate these, the larval and adult IPCs integrate a number of different inputs that modulate peptide expression and secretion. Many of these factors have been investigated in either larvae or adults, but not both (see Table 1). IPC regulation is known to differ between larvae and adult—e.g., in sugar sensitivity (above), and thus factors described here may or may not function similarly in adult and larval life. As mentioned above, information about the internal nutritional status following ingestion of food is sensed by the fat body, which relays this information to the IPCs in the brain via signals released into circulation. These adipokines include Eiger, the *Drosophila* Tumor Necrosis Factor Alpha (TNF-alpha) orthologue, which is released from larval fat-body cells under conditions of low internal amino-acid concentrations [51]. This signal acts through its receptor Grindelwald in the larval IPCs to activate the Jun Kinase cascade, leading to inhibition of DILP-gene expression. On the other hand, other larval nutrient-dependent fat-body signals such as CCHa2, Stunted, and GBP1/2 mediate positive actions on DILP production and release [49–51, 54, 179, 180]. The Activin-like factor Dawdle (Daw) is another IPC-modulating hormone, secreted by the larval fat body in response to the consumption of sugar [181]. Expression of *daw* is under the control of the carbohydrate response element binding protein (ChREBP) transcription factor Mondo-Mlx [182], and this hormone acts on the midgut to downregulate digestive enzymes after a sugary meal, a phenomenon called glucose repression that prevents acute nutritional overload [181]. Daw also promotes (likely indirectly) the release of insulin from the larval IPCs and regulates the expression of key metabolic enzymes of the tricarboxylic-acid (TCA) cycle [53]. Furthermore, neuronal populations that regulate energy storage are targets of Daw signaling, and ablation of these cells leads to starvation susceptibility due to lack of reserves [183]. Daw thus regulates energy absorption, storage, and use to maintain sugar homeostasis after intake. Fat-to-brain signaling via these various adipokines that regulate insulin signaling is, therefore, important to couple metabolism to the intake of nutrition. The CC is another source of IPC regulation. In the larva, high trehalose promotes Akh release, which appears to act on the IPCs to promote DILP3 release [139]. In the adult, at least, the CC also expresses the unrelated peptide Limostatin (Lst), which appears to be induced by sugar starvation [184]. The Lst receptor, LstR/PK1-R, is expressed in the adult IPCs and acts in these cells to reduce insulin release [184].

Furthermore, the IPCs also receive neuronal inputs via neuromodulators such as Leucokinin (Lk) [185]. In the adult, Lk is expressed in a set of neurons in the brain and nerve cord, and Lk/Lkr signaling appears to reduce adult DILP expression and release [186]. Lk also seems to coordinate behavioral responses with metabolic ones, since Lk also promotes adult food intake and locomotor activity [185] and regulates adult gustatory responses associated with the avoidance of bitter foods [187]. Taken together, these data fit a model in which Lk is a starvation-induced factor that acts to block insulin release, enhance the palatability of foods, and promote food-seeking and consumption behaviors to enhance animal survival under nutritionally poor environmental conditions. Pigment-dispersing factor (PDF), perhaps released synaptically from clock neurons onto IPC projections, also regulates adult IPC activity in response to circadian day-length stimuli, inhibiting insulin signaling and thus promoting the reproductively dormant diapause state under short-day conditions [188, 189].

Gut hormones also play key roles in metabolic adaptations and signal to a diverse set of target organs. Genetic, transcriptomic, and immunohistochemical evidence suggests that larval or adult midgut enteroendocrine cells express *AstA, *Allatostatin C (AstC), BursA, *CCHa1, *CCHa2, CNMamide (CNMa), Crustacean cardioactive peptide (CCAP), *Diuretic hormone 31 (Dh31), Ion-transport peptide (ITP), *Myoinhibitory peptide/Allatostatin B (MIP), Neuropeptide F (NPF), Neuropeptide-like precursor 2 (NPLP2, likely functioning as an apolipoprotein rather than, or in addition to, as a prepropeptide [190]), Orcokinin, *sNPF, and *Tachykinin (Tk), expressed in stereotyped combinations and anatomical regions [191–197]. However, without evidence of proper peptide processing and release, prepropeptide expression alone is insufficient to prove biological activity. Processed peptides from those prepropeptides marked with an asterisk have been identified in mass-spectrometric assays of the adult midgut [198]. Evidence for release of enteroendocrine peptides (processed or not) and downstream function has been reported for BursA [199–201], Dh31 [202, 203], NPF [204], and Tk [205]. Tk, either from neurons terminating near or on the IPCs or from the gut, activates its receptor TkR99D in the IPCs, where it is required for proper regulation of *DILP2* and *DILP3* expression [205, 206]. In the adult, loss of *TkR99D* in the IPCs leads to faster depletion of sugars under starvation and reduces survival under these conditions. Moreover, gut-derived Tk regulates gut lipid metabolism and overall lipid homeostasis in response to yeast feeding [205]. Tk also regulates aspects of starvation-induced modulation of sensory sensitivity [207]. Thus, this peptide is important for sensitivity to feeding cues, feeding drive, and proper utilization of the consumed materials. Furthermore, animals such as *Drosophila* need to modulate their metabolism and growth not only to nutrient conditions, but also to changing temperatures. Part of this response is mediated by cold-responsive thermosensory neurons that synapse directly upon the IPCs and regulate DILP expression and release to control larval growth according to changing temperatures [28].

### Signals that regulate the APCs

Akh expression appears to be tightly controlled, with similar peptide levels in animals carrying 1, 2, or 3 copies of the *Akh* genomic region [163]; furthermore, loss of the Akh peptide leads to increased *Akh* reporter-gene expression [159], suggesting that feedback inhibition occurs via AkhR either directly in the APCs or via intermediary cells. A handful of APC-exogenous hormonal and neuronal influences upon the APCs are known (Table 2), although there have been no reports of systematic attempts to identify these. Most of these influences are reported to act on both the APCs and the IPCs, and these are discussed in the next section. Only one APC-exogenous factor is reported to act on the APCs alone (indirectly): in the adult, gut-derived Bursicon-Alpha (BursA) acts via a neuronal relay to reduce Akh signaling during starvation [200]. However, several studies have been performed in the locust. In this insect, flight activity induces *Akh* expression [208] and peptide release to mobilize energy for long-distance travel [209]. Diverse small amines and peptides regulate the locust APCs [210–216], and it therefore seems likely that the regulation of the *Drosophila* APCs is rich and responsive to many behavioral and environmental stimuli as well.

### Signals that regulate both the IPCs and APCs to mediate nutritional adaptation

Under changing nutritional conditions, linking the regulation of energy uptake and release, mediated by the opposing effect of DILPs and Akh, through common nutritionally regulated mediators is important for maintaining homeostatic control. Several peptide hormones are known to act on both the IPCs and the APCs to promote homeostasis via the dual control of this regulatory circuit (see Fig. 2 and Tables 1, 2). In *Drosophila*, like mammals, the coordinated regulation of DILPs and Akh is key to adaptive responses to ingestion of different ratios of carbohydrate and proteins. While dietary sugar promotes insulin signaling and decreases Akh signaling to prevent hyperglycemia, ingestion of protein concomitantly increases both insulin and Akh to prevent insulin-induced hypoglycemia after protein-rich meals [217]. Thus, the coordinated regulation of DILPs and Akh maintains sugar homeostasis in response to varying dietary intake of sugar and protein. In larvae and adults, the neuropeptide receptor AstA-R2 is expressed in both the IPCs and APCs, suggesting that it regulates both DILP and Akh signaling. AstA and AstA-R2 are differentially regulated by consumption of sugars and protein, and this signaling system regulates feeding choices between these nutrients, promoting protein intake over sugar [78]. Activation of AstA-expressing neurons also inhibits the starvation-induced increase in gustatory sensitivity to sugar and blocks feeding [218]. Together these observations suggest that AstA is regulated by the dietary sugar-to-protein ratio and coordinates adaptive metabolic responses through regulation of DILPs and Akh.

Another peptide that has been shown to modulate both DILP and Akh signaling is sNPF, which is secreted from certain neurons of the brain in larvae and adults. In response to starvation, sNPF release upregulates feeding and DILP-gene expression (in anticipation of new nutrients) through the sNPF receptor (sNPF-R) in the IPCs, which is coupled to stimulatory G-proteins in these cells [219–224]. In a feedback arrangement, sNPF-positive neurons also express InR and, in response to DILP signaling, produce more sNPF to promote continued feeding. This feedback loop is required for the increase in feeding induced by short periods of starvation [223]. Other sNPF-expressing neurons of the adult brain sense hemolymph sugar and, under higher-sugar conditions, release peptide onto the IPCs and the APCs simultaneously [224]. In the IPCs, this is an activating stimulus that induces DILP release, while in the APCs, sNPF-R acts through inhibitory G-proteins, and, therefore, sNPF signaling blocks Akh release [224]. This peptide also regulates adult olfactory sensitivity, described below [225, 226]. Thus, in response to consumed sugars, this pleiotropic peptide coordinately raises insulin levels and lowers Akh levels, which promotes tissue uptake of hemolymph sugars and downregulates lipid-mobilizing processes [224], while also governing food-seeking behavior.

Insulin and Akh are also jointly controlled by Upd2. This protein is released by cells of the fat body in both larvae and adults in the fed state and acts through the receptor Domeless to inhibit certain GABAergic neurons of the brain, which synapse on the IPCs [21]. Upd2 signaling thus leads to derepression of the IPCs and promotion of insulin release in fed conditions. Furthermore, Upd2 is released from the adult musculature and acts on the APCs to govern Akh secretion and thereby to control lipid mobilization for energy use [122]. Thus, this signal is released from energy-storing and -consuming tissues and acts through both DILPs and Akh to coordinate metabolite storage, mobilization, and use.

### Hormonal control of lipid storage and release

Stored energy provides a buffer against times of scarcity or exertion. In nutrient-rich conditions, the fly sets aside excess energy in the form of TAG, stored within lipid droplets in the cells of the fat body. These stored lipids can be degraded and mobilized by metabolic enzymes such as lipases [227–229]. Among the most important fat-body lipases for metabolic adaptation is Brummer (Bmm), the *Drosophila* orthologue of mammalian adipose triglyceride lipase (ATGL) [230]. In the fed state, DILP signaling in the fat body via InR induces sugar uptake from the hemolymph and represses the expression of genes required for lipolysis [231–234]. Insulin signaling prevents FoxO activation of genes important for lipolysis, including *bmm* [234], and low Akh signaling allows expression of genes required for lipogenesis, such as *midway* [235]. High DILP activity and low Akh signaling thus gear the physiology of the fat body towards storage under fed conditions.

In lean times, hormonal influences including Akh/AkhR signaling induce the triacylglyceride-lipase-mediated breakdown of stored TAGs into DAGs in *Drosophila* [236]. The DAGs can then be transported in the hemolymph complexed with one of several lipid-carrier proteins [237]; alternatively, lipid components (fatty acids and glycerol) can be further broken down and reformed into trehalose through the process of gluconeogenesis (more specifically, trehaloneogenesis), reviewed elsewhere [238, 239]. In studied insects of a range of species, AkhR signaling passes through stimulatory G-proteins and has been shown directly to increase intracellular concentrations of cAMP and calcium [240–242]. Reports in *Drosophila* suggest that binding of Akh to AkhR may trigger an intracellular Ca^2+^ (iCa^2+^) second-messenger response via the G protein subunits Gαq and Gγ1 and phospholipase 21C (Plc21C) [235, 243]. Genetic experiments involving conditional knockdown of these downstream signaling components or the store-operated calcium entry (SOCE) component *Stim* lead to a blockage of fat-body Ca^2+^ entry and subsequent defects in organismal lipid mobilization [235, 243]. However, a direct demonstration of AkhR’s signaling mechanism in *Drosophila* through, e.g., ex-vivo fat-body calcium or cAMP quantification after Akh exposure has not been reported, to our knowledge.

In any case, second-messenger cascades initiated by AkhR signaling induce repression of the lipogenic gene *midway* and activate the expression of lipase genes, thereby blocking lipid synthesis while activating lipid breakdown [161, 232, 235]. This upregulation is aided by relief of DILP-induced inhibition [231, 232]. Together, in a fasting state, reduced DILP signaling and increased Akh activity switch the fat body into lipid-breakdown mode. The main intracellular sensor of nutrition (primarily amino acids), TOR, is also a component of lipid-metabolism regulation. Because insulin signaling and TOR are interlinked via Akt, TOR mediates some DILP-induced effects downstream of InR and also has effects of its own. Reduction of TOR activity in the fat body leads to smaller lipid droplets and reduced lipid storage [244]. Interestingly, TOR also regulates fat-body autophagy, a starvation-induced process that cells use to release and recycle store nutrients. In starved conditions, inactivation of TOR induces autophagy-mediated breakdown of nutrients, which can be released from the fat to sustain overall organismal survival under such conditions [245]. Through these mechanisms, fat-body intracellular nutritional levels thus also regulate lipid metabolism.

To provide greater control over lipid physiology, signals from other tissues modulate the AkhR signaling pathway in the fat body to gate lipid release. During development, at least, the TGF-β ligand Activin-β (Actβ) is secreted by endocrine cells of the gut and acts directly on cells of the fat body through its receptor Baboon (isoform A only) to regulate lipid metabolism and hemolymph sugar levels [170]. Baboon[A] signaling activates the downstream transcription factor dSmad2, which regulates *AkhR* expression, thereby adjusting fat cells’ sensitivity to the starvation-induced Akh signal. Chronic high-sugar feeding disturbs the balance of cell proliferation in the gut and leads to an increased number of Actβ-secreting cells; this extra Actβ induces abnormally high fat-body expression of *AkhR,* which triggers aberrant lipolysis and gluconeogenesis, thereby leading to carbohydrate imbalance and hyperglycemia [170].

However, the AkhR pathway, including modulators of its activity, is not the sole regulator of fat-body lipid mobilization. Additional, unidentified pathways appear to participate in the regulation of starvation-induced lipolysis in adipose tissue. Expression of Bmm lipase requires Akh signaling during short-term starvation (4 h) [232], but not over longer-term starvation, since fat-body *bmm* is upregulated even in *AkhR* mutants starved for 6 h [161]. Akh signaling during early starvation regulates lipases beyond Brummer, but Brummer is specifically required for later lipolysis [161]. Only in *AkhR bmm* double mutants is starvation-induced lipid mobilization fully suppressed, with identical lipid levels between fed flies and flies starved to death [161], suggesting the existence of other, uncharacterized signal(s) that regulate lipolysis through Bmm.

In addition to Actβ, the gut also secretes a lipid-associated form of the protein Hedgehog (Hh) under starvation conditions. This signal promotes lipid mobilization in the fat body in both larvae and adults and supports hemolymph sugar levels, but only in starved animals, indicating the requirement for other permissive mobilization signal(s) [94, 246]. Recent work shows that Hh acts on the fat to upregulate *bmm* expression. Furthermore, the sugar-induced gut-secreted factor BursA [200] may also act on the fat body. Burs dimers activate the transcription factor Relish, the *Drosophila* orthologue of mammalian NF-κB, in the fat body. This activates innate-immunity pathways to prevent infection during these transitions [247]. Relish also antagonizes FoxO-induced *bmm* expression to limit fasting-induced lipolysis [248]. Investigating the emerging link between immune response and metabolism will be an important direction for future research. Furthermore, characterizing the signals that affect the fat will be key to the understanding of lipolytic control and the mobilization of resources in the face of environmental and nutritional challenges.

### Mobilization of glycogen stores

As in other multicellular organisms, the polysaccharide glycogen is the main storage form of carbohydrates in *Drosophila* [249]. In both the larval and adult stages, glycogen is synthesized and stored in several tissues including the central nervous system (CNS), fat body, and skeletal muscles, and the dynamic regulation of glycogen metabolism—especially during starvation—plays a key role in maintaining metabolic homeostasis [124, 126]. For example, glycogen stores in larval body-wall muscles and fat body, but not CNS, are rapidly depleted during larval starvation, suggesting that glycogen mobilization is differentially regulated between organs, and that especially the fat body acts as an important carbohydrate reservoir buffering circulating energy levels [126, 250]. Similarly, although glycogen appears to be largely dispensable for adult fitness under fed conditions, muscle glycogen is a crucial factor in maintaining stereotypic locomotor activity and wing-beat frequency during starvation [250, 251], indicating that glycogen metabolism is regulated in both a tissue- and stage-specific manner. Glycogen metabolism is controlled by two enzymes, glycogen synthase (GlyS) and glycogen phosphorylase (GlyP), the latter of which catalyzes the rate-limiting step in glycogen breakdown. The control of these processes appears to depend largely on hemolymph sugar levels, and they are generally regulated organ-autonomously rather than by systemic signals such as Akh [126]. The systemic stress peptide Corazonin (Crz) and its receptor CrzR—paralogues of Akh and AkhR [173, 252, 253]—may regulate glycogen content of the adult fat body [254]. Knockdown of CrzR using transgenes targeting this tissue does not affect lipid metabolism but does increase glycogen stores [254]; however, the authors do not rule out these transgenes also target the salivary glands, which also express CrzR and are also involved in energy balance via production of feeding-related enzymes and fluids [254]. Furthermore, glycogen breakdown is also regulated by autophagy-dependent mechanisms, at least in skeletal muscle, and genetic experiments reveal that both mechanisms are necessary for maximal glycogenolysis. Interestingly, GlyS may be a central regulator of both pathways via its direct interaction with Atg8, hereby linking glycogenolytic activities with glycogen autophagy to homeostatically control glycogen breakdown in flies [255].

### Circadian rhythms of metabolism

The adult fly is exposed to the daily cycling of the ambient photic and thermal environment, which brings both opportunity (finding food sources and mates) and danger (predation and desiccation). To anticipate these cycles and schedule appropriate behavior and physiology, flies possess a central neuronal circadian clock that governs rhythmic behaviors such as feeding and sleeping (Fig. 2). This review focuses on metabolic rhythms; an excellent general review of *Drosophila* circadian rhythm has recently been published [256].

The adult IPCs are synchronized with the internal circadian clock via synaptic connections, with greater IPC electrical activity in the subjective morning; however, feeding animals at night, when the IPCs are normally quiet, induces morning-like electrical activity in these cells [257]. The IPCs also express receptors for PDF, the main output factor of the clock, and for sNPF, which is co-expressed in certain PDF-expressing cells [188]; these inputs also connect circadian rhythms to the IPCs, and they appear to be part of a diapause-antagonizing system as well. Daily activity regulates Akh signaling as well, via the cytokine Upd2 [122]. Thus, circadian information is integrated into metabolic programming.

Beyond the central-brain clock that drives systemic signaling, scattered peripheral intracellular oscillators regulate local processes (Fig. 2). One such peripheral clock governs fat-body physiology [258]. Flies lacking this clock eat more than controls, especially at night, and are sensitive to starvation, due to low glycogen levels, indicating a loss of proper energy storage regulation [258]. The adult gut also exhibits endogenous circadian oscillation in gene expression and cell proliferation [259, 260]. As a result of circadian rhythm-driven changes in physiology, metabolite levels also vary in a circadian fashion: in a recent study, 14% of metabolites were seen to vary in abundance with a daily rhythm, and ~ 64% of these were observed to cycle even under constant darkness [261].

Local oscillators also participate in behavioral governance. Olfactory receptor neurons (ORNs) express their own clock systems, leading to cyclical patterns in the amplitude of odor responses [262–264]. These patterns of antennal response translate into cyclical odor-driven behavioral patterns [265]. Likewise, gustatory receptor neurons (GRNs) display cyclical patterns of electrophysiological responses to tastants, and this cyclicity translates into circadian rhythms of behavioral response to tasted compounds [266]. Abolishing the clock in these GRNs mimics starvation and leads to overeating and increased metabolite stores [266].

### Adaptive modulation of feeding behaviors

In changing environmental conditions, the location and quality of food sources are dynamic. Flies are attracted by certain chemicals in the food while being repelled by other cues that represent potential danger. *Drosophila* sense the positive and negative qualities of potential food sources through taste and smell and will initially avoid marginal sources. When nutritional balance is low, flies exhibit several stereotypical behavioral changes that increase their ability to find new sources of food, as well as make them more amenable to consuming marginal or dangerous food. They become more active, they sleep less, and they adjust their senses of olfaction (chemosensation of airborne chemicals—“smell”) and gustation (chemosensation by contact, or “taste”). It is thought that increased locomotor activity increases the chances that a fly will encounter a food source, and adjustment of sensory sensitivity makes a fly both more likely to be attracted to weak food odors and less likely to be repelled by noxious ones. Feeding regulation in the fly has been intensively researched, identifying a broad array of factors governing food-related behaviors. We cover here adaptive feeding responses regulated by DILPs and Akh (Fig. 2), although many other factors have been characterized, including AstA [78, 218, 267], Dh44 [17, 18], Hugin [268–272], Lk [185, 186, 273], NPF [274–276], sNPF [220, 222, 277, 278], and members of the TGF-β family [53, 170, 181, 183]. The general regulation of feeding is reviewed comprehensively elsewhere [178, 279, 280]. Through these and other changes, the starved fly becomes more likely to be able to survive, although at the risk of toxicity or exhaustion.

### Starvation-induced hyperactivity

Akh signaling is essential for the phenomenon of starvation-induced hyperactivity, thought to represent an adaptive food-seeking behavioral response to nutritional deprivation. Hypotrehalosemia-induced Akh release triggers starvation-induced hyperactivity, including during periods normally characterized by inactivity or sleep [160, 281]. This response is induced by Akh/AkhR signaling in certain octopaminergic neurons of the brain [282, 283]. Octopamine is generally considered the insect analogue of noradrenaline, and it acts through several receptors in many cells to increase arousal. Interestingly, these AkhR-expressing octopaminergic neurons also express InR, whose activation by DILPs inhibits their signaling [282]. Thus, when sugar is low, Akh acts to increase arousal via these octopaminergic cells, which promotes wakefulness and locomotor activity as a way to find food; then, when food has been consumed, the increase in hemolymph sugar induces DILP release, which terminates the excitatory octopamine signal and thus promotes quiescence.

### Modulation of olfaction by nutritional status

Olfaction, which detects chemical signals from potentially remote sources, is an important component of food-seeking behavior and adaptation to dynamic environments (Fig. 2). Under fasted situations, animals’ acuity for appetitive odors is heightened, and their behavioral response to them is increased, enabling them to be drawn towards weaker or more distant sources of odor plumes [284], which represent potential food sources. At the same time, sensitivity to, and avoidance of, aversive odors—those that represent potential toxicity or danger—is decreased, allowing the animal to be attracted to riskier food sources. These processes are induced by hormonal signals that reflect the nutritional status of the animal as well as other signals related to the internal and external state.

Olfaction is mediated by olfactory receptors (ORs) stereotypically expressed in identifiable olfactory receptor neurons (ORNs); the neuroanatomy and odor-responsiveness of this system has been very well mapped [285]. These receptors and neurons are generally grouped into two behavioral classes: appetitive (attractive) and aversive (repellent). The appetitive ab1a ORNs, which express the fruit-ester-sensitive OR42b, are required for olfactory-guided food-searching behavior [225]. These cells are directly made more active under low-nutrient conditions via the action of sNPF signaling [225]. Starvation induces the expression of sNPF-R in the ab1a ORNs to increase their sensitivity to attractive odors [225]. When fed conditions return, nutritional intake induces DILP release, which downregulates *sNPF-R* expression in the ab1a ORNs via InR signaling, reducing these sensory neurons’ excitability [225]. About a quarter of ORNs express sNPF-R [226]; given the ability of this receptor to either activate or inhibit neurons [224], many odorant responses may be up- or down-regulated by this mechanism. Thus, low nutrition upregulates appetitive responses to increase food-seeking success, and once a food source is found and the internal nutritional state returns to normal, sensitivity is downregulated again, to prevent unneeded attraction to odors.

Another class of adult appetitive ORNs, the ab3A neurons that express the ester-detecting odorant receptor 22a, express NPFR and are thus regulated by NPF https://pubmed.ncbi.nlm.nih.gov/28476120/. In fed conditions, the brain produces the satiety signal Unpaired-1 (Upd1), which inhibits the NPF-releasing cells of the brain [286]. In poor conditions, these cells are derepressed, leading to the release of NPF [286]. Among the many feeding-promoting effects of NPF is the increase in sensitivity of the ab3A neurons. While heightening the animal’s sensitivity to appetitive odors, fasting simultaneously reduces the fly’s sensitivity to aversive odors, allowing fasted flies to be attracted to sites they might normally avoid. Tk and one of its receptors, TkR99D, act in sensory neurons expressing the aversive receptor OR85a to inhibit them under starvation [207]. Through this and similar neuromodulators, animals’ sensitivity to noxious odors, which represent toxicity or danger and tend to repel flies, is reduced, which allows them to be drawn to risky food sources. In addition to these characterized pathways by which hunger modulates adult olfactory sensitivity, the satiety peptide Dsk appears to reduce larval olfactory sensitivity to attractive odors [154]. This means that multiple hormonal systems act on sensory neurons to increase animals’ attraction to appetitive stimuli and simultaneously function to reduce the aversive effects of noxious odors, broadening the range of odor concentrations that the fly will be drawn to. This allows the starved animal to find less-nutritious food, which it otherwise would not find attractive.

### Modulation of gustation by nutritional status

Like olfaction, which allows an animal to find a distant food source, gustation is an integral part of feeding behavior. When flies are in a non-starved state, they will consume only foods they perceive to be highly nutritious (e.g., sweet or protein-rich foods) with low concentrations of toxic compounds, which are perceived as aversive. As hemolymph sugar drops, flies become more likely to consume foods of poor quality, balancing the risk of death by starvation against the risk of being poisoned by low-quality or toxic food. This change is brought about by modulating the flies’ gustatory sensitivity both to nutritional compounds and to potential toxins.

Flies carry gustatory receptor neurons (GRNs) on various external surfaces; among these are the tarsi (“feet”) and legs, allowing them to taste the surfaces they walk on, while GRNs on the proboscis allow tasting of food at consumption. GRNs express gustatory receptors (GRs) tuned to a variety of chemical classes, including sugars, salts, and potentially toxic bitter compounds. Like ORs and ORNs, GRs and GRNs have either appetitive or aversive valence, and like those olfactory components, the gustatory system is also subject to sensitivity-adjusting neuromodulation in response to nutritional sufficiency or deficiency (Fig. 2). In low-sugar states, Akh is released into the hemolymph from the APCs, and among its functions is the modulation of gustatory sensitivity. Adult sweet-sensing (Gr5a^+^, appetitive) GRNs express AkhR, and Akh signaling under fasting conditions increases the excitability of these neurons, thus inducing flies to feed on foods that offer low levels of nutrition that would be ignored under better nutritional conditions [162]. Starvation also lifts Upd1’s inhibition of NPF signaling, which leads to NPFR-induced excitation of dopaminergic neurons contacting the Gr5a^+^ GRNs, and increased dopamine signaling further enhances the animal’s sugar sensitivity [287]. Through these actions, the fly becomes increasingly likely to be triggered to feed by low levels of sugar in the food source. In parallel, aversive GRNs are inhibited under fasting conditions by sNPF and Akh [287] and NPF [288]. This indicates that starvation increases the perceived palatability of food by several routes. Dsk released from the IPCs in the fed state is also required for the inhibition of consumption of unpalatable food [131], although the hierarchical level at which it acts—through regulation of gustation, higher-level gustatory processing and integration, or feeding motivation, for example—is unknown.

## Concluding remarks

The developmental and metabolic demands placed on *Drosophila*, and their responses to these, are complex and dynamic, as illustrated above. Larvae optimize development to produce the most reproductively successful adults that conditions will allow. To do this, they adjust their growth rate and growth duration by regulating intracellular and systemic growth factors such as TOR, insulin, PTTH, and ecdysone. We propose that the IPCs, PTTHn, and the PG are signaling hubs that integrate environmental cues to coordinate growth rate and duration to adjust final size in response to given conditions. Because of the strong conservation between mammalian and insect hormonal systems such as insulin-like signaling, growth- and steroid-hormone pathways, and peptide neuromodulation, studies of these aspects of *Drosophila* can provide important frameworks for understanding the link between environmental factors and disorders including diabetes and obesity. The mechanistic bases of how animals assess the critical-weight checkpoint is unresolved and is a key direction for future research. In *Drosophila* and mammals, including humans, “critical weight” may correspond to a certain amount of adiposity. Insights from *Drosophila* into nutrition-dependent developmental checkpoints have the potential to illuminate mammalian size regulation, including the molecular mechanisms underlying the link between childhood obesity and early puberty.

*Drosophila* also regulates its metabolism according to prevailing conditions, and this includes behavioral responses, such as feeding decisions. Central to both these metabolic and behavioral changes are the insulin and Akh systems, which regulate numerous downstream systems to modify metabolic pathways and feeding decisions. Intertwined with these and other hormonal systems, gustatory and olfactory systems also play important roles in regulating the interface between the organism and the environment. The inter-organ signaling networks that function upstream of insulin and Akh need to be explored systematically to further understand how organisms adapt metabolism to environmental conditions. While much is known about insulin regulation, the mechanisms underlying Akh regulation and energy mobilization from adipose tissue are important but largely unresolved questions. Regulation imposed by the counter-regulatory actions of insulin and Akh are key to maintaining metabolic homeostasis in variable environments. Studies in *Drosophila* will undoubtedly continue to reveal new mechanistic insights into animal metabolic regulation.
